# Progressive Retinal Degeneration and Glial Activation in the CLN6*^nclf^* Mouse Model of Neuronal Ceroid Lipofuscinosis: A Beneficial Effect of DHA and Curcumin Supplementation

**DOI:** 10.1371/journal.pone.0075963

**Published:** 2013-10-04

**Authors:** Myriam Mirza, Cornelia Volz, Marcus Karlstetter, Monica Langiu, Aleksandra Somogyi, Mika O. Ruonala, Ernst R. Tamm, Herbert Jägle, Thomas Langmann

**Affiliations:** 1 Institute of Human Genetics, University of Regensburg, Regensburg, Germany; 2 Department of Ophthalmology, University of Cologne, Cologne, Germany; 3 Department of Ophthalmology, University of Regensburg, Regensburg, Germany; 4 Center for Membrane Proteomics, University of Frankfurt am Main, Frankfurt am Main, Germany; 5 Institute of Human Anatomy and Embryology, University of Regensburg, Regensburg, Germany; National Eye Institute, United States of America

## Abstract

Neuronal ceroid lipofuscinosis (NCL) is a group of neurodegenerative lysosomal storage disorders characterized by vision loss, mental and motor deficits, and spontaneous seizures. Neuropathological analyses of autopsy material from NCL patients and animal models revealed brain atrophy closely associated with glial activity. Earlier reports also noticed loss of retinal cells and reactive gliosis in some forms of NCL. To study this phenomenon in detail, we analyzed the ocular phenotype of CLN6*^nclf^* mice, an established mouse model for variant-late infantile NCL. Retinal morphometry, immunohistochemistry, optokinetic tracking, electroretinography, and mRNA expression were used to characterize retinal morphology and function as well as the responses of Müller cells and microglia. Our histological data showed a severe and progressive degeneration in the CLN6*^nclf^* retina co-inciding with reactive Müller glia. Furthermore, a prominent phenotypic transformation of ramified microglia to phagocytic, bloated, and mislocalized microglial cells was identified in CLN6*^nclf^* retinas. These events overlapped with a rapid loss of visual perception and retinal function. Based on the strong microglia reactivity we hypothesized that dietary supplementation with immuno-regulatory compounds, curcumin and docosahexaenoic acid (DHA), could ameliorate microgliosis and reduce retinal degeneration. Our analyses showed that treatment of three-week-old CLN6*^nclf^* mice with either 5% DHA or 0.6% curcumin for 30 weeks resulted in a reduced number of amoeboid reactive microglia and partially improved retinal function. DHA-treatment also improved the morphology of CLN6*^nclf^* retinas with a preserved thickness of the photoreceptor layer in most regions of the retina. Our results suggest that microglial reactivity closely accompanies disease progression in the CLN6*^nclf^* retina and both processes can be attenuated with dietary supplemented immuno-modulating compounds.

## Introduction

Neuronal ceroid lipofuscinoses (NCL) are a group of inherited progressive neurodegenerative lysosomal storage disorders with a frequency of 7–8 in 100,000 live births worldwide [1], [2]. Mutations in at least thirteen CLN genes give rise to different forms of NCL with different onset and clinical course of the disease [3]–[5]. A general hallmark of all NCL subtypes is the accumulation of autofluorescent material in neurons causing progressive degeneration and tissue atrophy [6]. This results in clinically common features shared by all NCL disorders, including visual impairment, mental and motor deficits, spontaneous seizures and premature death [7]. The ocular pathology in patients with the infantile type of NCL showed a complete disappearance of photoreceptors, bipolar cells and ganglion cells of the retina along with a marked reactive gliosis also indicating severe retinal degeneration and glial reactions in some forms of NCL [8].

The CLN6 gene encodes a transmembrane protein of unknown function and mutations cause the variant-late infantile form of NCL (vLINCL) as well as an adult form termed Kufs disease [9]–[11]. A naturally occurring mouse model CLN6*^nclf^* contains a frameshift truncation in both nclf genes [9]. Histological studies in the brain of these mice showed progressive astrocyte activation and microgliosis [12], [13] but little is known about the ocular phenotype and immune responses in the retina. Glial activation has also been described in the brain of a CLN6*^nclf^* sheep model [14] as well as other NCL models [15], indicating that inflammation and glial processes are another hallmark of NCL. However, it is currently unknown whether the modulation of microglial response can affect disease progression.

There is a growing interest in the identification of natural compounds to limit neuroinflammation and simultaneously support neuronal survival [16]. Among the naturally occurring immunomodulators, curcumin ((E,E)-1,7-bis(4-hydroxy-3-methoxyphenyl)-1,6-heptadiene-3,5-dione), a major constituent of tumeric, inhibits the defense program of microglia by diminishing the production of nitric oxide and secretion of proinflammatory cytokines [17], [18]. Docosahexaenoic acid (DHA, 22∶6n-3), a polyunsaturated fatty acid enriched in fish oil also dampens microglial nitric oxide production [19] and attenuates microglial reactivity in a mouse model of inherited retinal degeneration [20].

For the purpose of our studies, we used the CLN6*^nclf^* mouse retina as a model to study disease progression and therapeutic effects of immunomodulatory compounds. We comprehensively characterized the retinal degeneration of these mice using histology, immunohistochemistry, optokinetic measurements, electroretinography and glial marker expression. These analyses identified a progressive early retinal degeneration in CLN6*^nclf^* mice coinciding with prominent microglial and Müller cell response. In a therapy study, CLN6*^nclf^* mice supplemented with curcumin or DHA showed improved retinal function as well as attenuated microglial reactivity. These data suggest that immuno-modulatory compounds may play a protective role in NCL.

## Materials and Methods

### Animals

Wild-type and CLN6*^nclf^* mice were all on a C57BL/6J background. CLN6*^nclf^* mice and control mice were kindly provided by Prof. Klaus Rüther (Sankt Gertrauden-Krankenhaus Berlin). Animals were maintained in an air-conditioned environment on a 12-hour light–dark schedule at 22°C, and had free access to food and water. The health of the animals was regularly monitored, and all procedures were approved by the University of Regensburg animal rights committee and complied with the German Law on Animal Protection and the Institute for Laboratory Animal Research Guide for the Care and Use of Laboratory Animals, 2011. Animals were tested for the presence of the rd8/Crb1 mutation as described previously [21].

### Microscopy

Before enucleation, eyes were branded on the superior limbus. Eyes were fixed for 24h in Ito’s fixative and embedded in Epon (Serva, Heidelberg, Germany). Sections 1 µm in thickness were cut along the nasal-temporal plane and stained with fuchsin/methylene blue for morphometric analyses using light microscopy. Immunohistochemial analyses were performed on 10 µm retinal sections embedded in optimal cutting temperature (OCT) compound (Hartenstein, Wuerzburg, Germany) or retinal flat mounts. Samples were fixed in 4% paraformaldehyde, rinsed and rehydrated with PBS. Sections were blocked with a dried milk solution followed by an overnight incubation with primary antibodies at 4°C. Antibodies included rabbit anti-Iba1 antibody (diluted 1∶500; Wako Chemicals, Neuss, Germany) and rabbit anti-GFAP antibody (diluted 1∶600; Sigma-Aldrich). After washing, samples were labeled with a secondary antibody conjugated to Alexa488 (Jackson Immuno-Research, West Grove, PA, USA) and counter-stained with DAPI. Sections were mounted in DAKO fluorescent mounting medium (Dako Cytomation GmbH, Hamburg, Germany) and viewed with Axioimager Z1 Apotome Microscope (Carl Zeiss, Goettingen, Germany). Flat mounts were mounted and viewed with Axioimager Z2 Apotome Microscope (Carl Zeiss, Goettingen, Germany) using z-stacks of inner and outer plexiform layers as indicated by fluorescent sidebars. The microglial phenotype in wild-type and food supplemented CLN6*^nclf^* mice was determined by quantification of ramified and amoeboid microglial cells in nine different flat mount areas.

### Behavioral Studies

Optokinetic tracking was assessed as a predictor of visual acuity using a virtual optomotor system (Optomotry, Cerebral Mechanics, Lethbridge, Alberta, Canada) as described previously [22], [23]. Briefly, freely moving animals were exposed to moving sine wave gratings of various spatial frequencies and reflexively tracked the gratings by head movements. An automated staircase paradigm adjusted the spatial frequency of the rotating pattern on subsequent trials until a threshold was achieved. The OKT threshold was defined as the highest spatial frequency obtained at 100% contrast.

Rotarod experiments assessing motor neuron and cognitive difficulties were performed on age-matched wild-type and CLN6*^nclf^* mice using an accelerating Rotarod (PanLab/Harvard Apparatus, Holliston, MA). The rotarod started at 4 rpm and accelerated to 40 rpm over 60 seconds. The latency time to fall off was determined. Experiments were performed three times for each mouse with 15 minute resting time in between. The same experiment was carried out on two subsequent days to have a training effect. The latency time was then normalized to day 1 wild-type animals.

### Electroretinography

Mice were dark adapted for at least 12 hours before the experiments and subsequently anesthetized by subcutaneous injection of ketamine and xylazine. Pupils were dilated with tropicamide eyedrops (Mydriaticum Stulln; Pharma Stulln, Germany). Silver needle electrodes served as reference (fore-head) and ground (tail) and gold wire ring electrodes as active electrodes. Corneregel (Bausch & Lomb, Berlin, Germany) was applied to keep the eye hydrated and maintain good electrical contact. ERGs were recorded using a Ganzfeld bowl (Ganzfeld QC450 SCX, Roland Consult, Brandenburg, Germany) and an amplifier & recording unit (RETI-Port, Roland Consult, Brandenburg, Germany). ERGs were recorded from both eyes simultaneously, band-pass filtered (1 to 300 Hz) and averaged. Single flash scotopic (dark adapted) responses to a series of ten LED-flash intensities ranging from −3.5 to 1.0 log cd.s/m^2^ with an inter stimulus interval of 2 s up to 20 s for the highest intensity were recorded. Response waveforms were analyzed by means of through and peak amplitude and implicit time measurement. All analysis and plotting was carried out with R 2.15.2 and gplot 0.9.2.

### RNA Isolation and Reverse Transcription

Total RNA was extracted from total retina according to the manufacturer’s instructions using the RNeasy Mini Kit (Qiagen, Hilden, Germany). Purity and integrity of the RNA was assessed on the Agilent 2100 bioanalyzer with the RNA 6000 Nano LabChip® reagent set (Agilent Technologies, Boeblingen, Germany). The RNA was quantified spectrophotometrically and then stored at −80°C. First-strand cDNA synthesis was performed with RevertAid™ H Minus First Strand cDNA Synthesis Kit (Fermentas. St-Leon-Roth, Germany).

### Quantitative Real-time RT-PCR

Amplifications of 50 ng cDNA were performed with an ABI7900HT machine (Applied Biosystems, Darmstadt, Germany) in 10 µl reaction mixtures containing 1×TaqMan Universal PCR Master Mix (Applied Biosystems), 200 nM of primers and 0.25 µl of dual-labeled probe (Roche ProbeLibrary, Roche Applied Science, Mannheim, Germany). The reaction parameters were as follows: 2-min 50°C hold, 30-min 60°C hold, and 5-min 95°C hold, followed by 45 cycles of 20-s 94°C melt and 1-min 60°C anneal/extension. Measurements were performed in duplicates. Results were analyzed with an ABI sequence detector software version 2.3 using the ΔΔCt method for relative quantification and ATP5B as stable reference gene [24]. A Ct (cycle threshold) value of 35 was used as a cutoff for estimating significantly expressed transcripts. Primer sequences and Roche Library Probe numbers are listed in Table 1.

**Table 1:** Primer pairs and Roche library probes used for real-time qRT-PCR.

Gene	F-Primer (5′-3′)	R-Primer (5′-3′)	Probe
ATP5B	ggcacaatgcaggaaagg	Tcagcaggcacatagatagcc	77
C1Qa	ggagcatccagtttgatcg	Catccctgagaggtctccat	16
CD95	aaaccagacttctactgcgattct	Gggttccatgttcacacga	76
EDN2	tggcttgacaaggaatgtgt	Gccgtagggagctgtctgt	29
EGR1	ccttccagggtctggagaa	Actgagtggcgaaggcttta	3
GFAP	acagactttctccaacctccag	Ccttctgacacggatttggt	64
NCLF	ggcgaagaaggtgaagatga	Agagccacatgccaggac	104

### Supplementation Study

Experimental mouse diets were ordered from SSNIFF Spezialdiäten GmbH (Soest, Germany) consisting of standard mouse diet EF-M (control) without (control) or with either 0.6% Curcumin (99% Pure, ChemHome, Shanghai Honghao Chemicals Co.,Ltd., Shanghai, China) or 5% DHA (DHASCO-T containing 40% DHA, Martek Biosciences Corporation, Columbia, MD, USA). Mice (n = 12) started receiving supplement diets immediately after weaning (post natal day 21–23) for the next 30 weeks. Body weights were measured on a weekly basis for the duration of the study and found to be similar between supplementation and control groups.

### Statistical Analyses

RT-PCR data from different mouse ages, quantification of retinal layer thickness, optokinetic tracking results and ERG experiments were analyzed using a two-way ANOVA with Bonferroni post-test. Rotarod experiments were analyzed using a Kruskal-Wallis ANOVA. Microglia quantification was analyzed with an unpaired two-tailed *T*-test. *P*≤0.05 was considered as statistically significant.

## Results

### Progressive Degeneration, Lipofuscin Accumulation, and Glial Activation in the CLN6^nclf^ Retina

**Figure 1:**
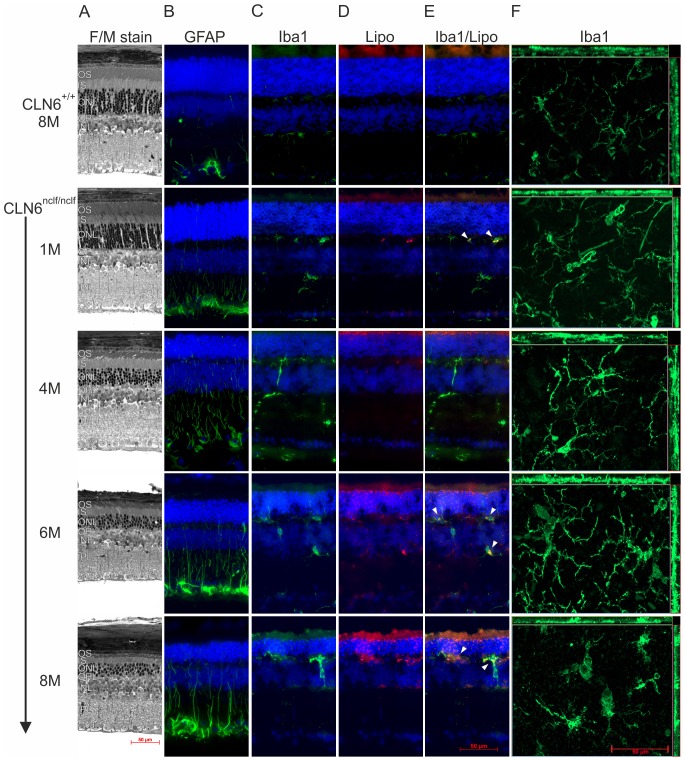
CLN6*^nclf^* mice show progressive degeneration, lipofuscin accumulation, and microglial reactivity in the retina. A. Histological changes in retinal sections from 8 month old wild-type mice compared to different ages of CLN6*^nclf^* mice using fuchsin/methylene blue staining. B. Immunolabeling of reactive Müller cells in wild-type and aging CLN6*^nclf^* retinas using anti-GFAP antibody. C. Staining of microglial cells with anti-Iba1 antibody. D. Autofluorescent lipofuscin accumulation in wild-type and CLN6*^nclf^* retinas. E. Merged images of anti-Iba1 immunolabelling with autofluorescent lipofuscin deposits. Arrow heads indicate co-localization of lipofuscin with amoeboid microglial cells. F. Anti-Iba1 labeled retinal flat-mounts reveal different microglial morphologies in wild-type and CLN6*^nclf^* retinas. The thickness of the flat-mount is indicated on the sides of the images. OS, outer segments; IS, inner segments; OPL, outer plexiform layer; INL, inner nuclear layer; IPL, inner plexiform layer; GCL, ganglion cell layer. Scale bar, 50 µm.

To characterize the temporal retinal degeneration and glial activation in CLN6*^nclf^* mice we studied animals at different ages ranging from one to eight months. Histological analyses showed a progressive degeneration of all retinal layers in CLN6*^nclf^* mice compared to wild-type controls (Fig. 1A). At eight months of age, the photoreceptor cell layer in particular was severely compromised in CLN6*^nclf^* retinas with only a few rows of cell nuclei remaining (Fig. 1A). We next used GFAP to assess the Müller glial status in the retina of control and CLN6*^nclf^* mice. While wild-type eight month old mice had some filamentous but mostly end-feet GFAP and/or astrocyte staining, CLN6*^nclf^* mice had a markedly increased GFAP expression as indicated by intensely stained filamentous structures spanning all retinal layers starting at one month of age (Fig. 1B). Increased GFAP expression at early ages in CLN6*^nclf^* mice indicates reactive Müller cell gliosis as a prominent early event in retinal degeneration. Retinal sections were also stained with the microglia marker Iba1 to assess changes in microglial morphology and migration into different layers (Fig. 1C). Resident, non-alerted microglial cells usually reside in a ramified form in both plexiform layers as seen in the wild-type control (Fig. 1C). In contrast, one month old CLN6*^nclf^* retinas already had amoeboid microglia cells with protrusions reaching into the nuclear cell layers. This effect was even more prominent in four, six, and eight month old CLN6*^nclf^* retinas with bloated microglia infiltrating the nuclear layers. The lipofuscin deposits visible in photoreceptor and inner-retinal layers of one month old CLN6*^nclf^* mice constantly increased in size and number with age (Fig. 1D). Interestingly, the colocalization of autofluorescent lipofuscin deposits with alerted phagocytic microglial cells suggests that these cells phagocytose significant amounts of storage material (Fig. 1E, arrow heads). To further confirm the morphological transition of ramified microglia cells into large phagocytes retinal flat-mounts were stained with Iba1. While in retinas from wild-type mice a highly ramified microglia network was evident (Fig. 1F) those from one month old CLN6*^nclf^* retinas already showed a mixed population of ramified and phagocytic microglia. As the mice aged, the cells became rounder in shape with shorter protrusions, indicating a loss of the ramified network structure and an altered state (Fig. 1F).

**Figure 2:**
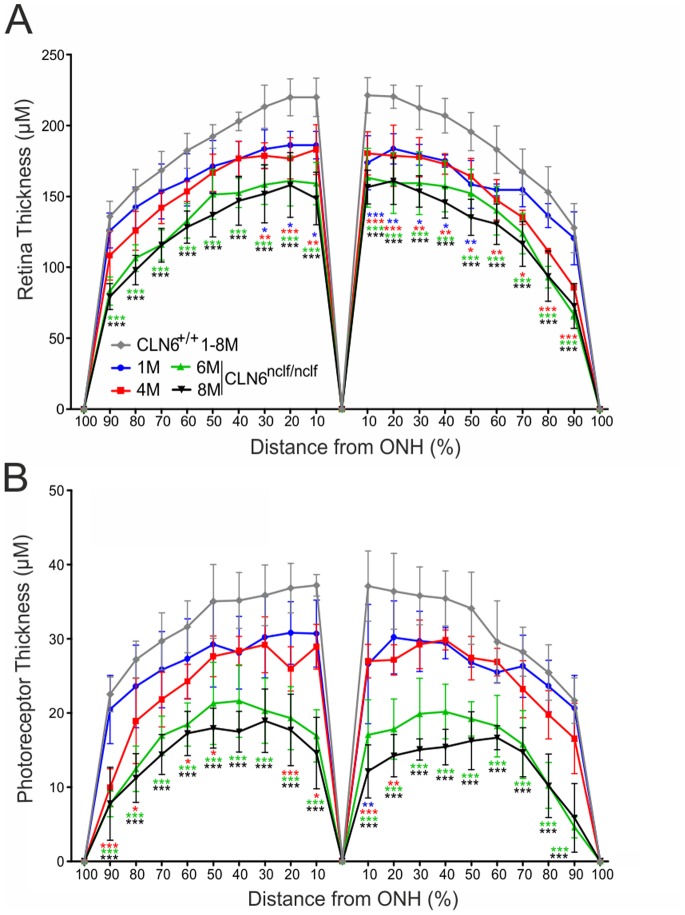
Age-dependent thinning of retinal and photoreceptor layers in CLN6*^nclf^* retinas. Anterior and posterior retinal areas were divided into ten sections with the optic nerve head as reference. A. Quantification of whole retinal thickness of CLN6*^nclf^* retinas compared to wild-type controls (mean ± SD). B. Quantification of photoreceptor layer thickness compared to wild-type controls (mean ± SD). *p<0.05; **p<0.01; ***p<0.001 CLN6*^nclf^* vs. age-matched wild-type mice, n = 4 animals per age group, two-way ANOVA followed by Bonferroni post-test.

In order to quantify the retinal degeneration observed, morphometric analyses of the whole retina and the photoreceptor layer were performed. Whole retinal measurements indicated that changes occurred in the central retina already in one month old CLN6*^nclf^* mice, an effect that spreads steadily with disease progression across the whole retina (Figure 2A). Of note, a significant decrease in thickness of the photoreceptor layer became evident at four months and thus correlates with the changed microglia phenotype (Figure 2B).

### Progressive Visual Decline in Aging CLN6^nclf^ Mice

**Figure 3:**
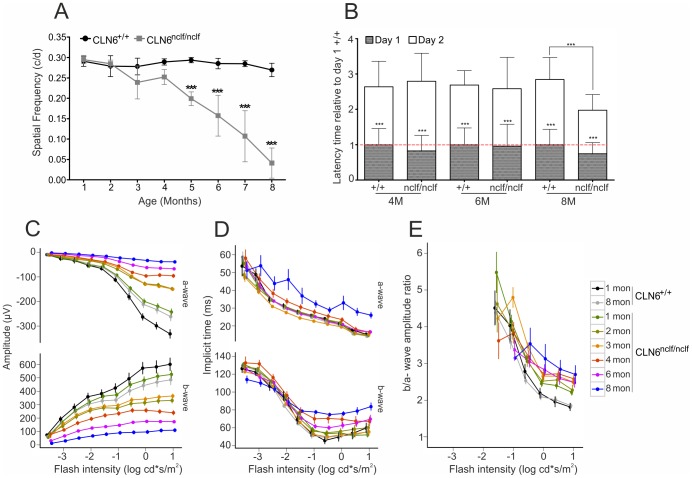
Aging CLN6*^nclf^* mice show a progressive reduction in visual function which is independent from motor deficits. A. Temporal changes in optokinetic tracking thresholds (cycles/degree) in CLN6*^nclf^* and wild-type mice ± SD. ***p<0.001, n = 12 animals per age group, two-way ANOVA followed by Bonferroni post-test. B. Rotarod performance of wild-type and CLN6*^nclf^* mice aged 4, 6 and 8 months. Data was normalized to the performance of day 1 wild-type mice ± SD. ***p<0.001, n = 6–15 animals per age group, Kruskal-Wallis ANOVA. C-E. Dark adapted (scotopic) ERG response amplitudes, implicit times and b/a-wave amplitude ratios of age-matched wild-type and CLN6*^nclf^* mice. Each symbol represents the mean of three animals ± SEM. For the brightest flash intensity, mean amplitude values of CLN6*^nclf^* mice and age-matched controls were compared with ANOVA. a-wave: 1 month: *p* = 0.0048, 2 months: *p* = 0.0005, 3 to 8 months: *p*<0.0001. b-wave: 1 month: *p* = 0.245, 2 months: *p* = 0.059, 3 to 8 months: *p*<0.0001.

We next studied the visual performance of CLN6*^nclf^* mice using optokinetic tracking (OKT). OKT is a good predictor of visual acuity when measuring reflexive head tracking to moving gratings using stairway changes in spatial frequency. Wild-type mice had a stable maximal OKT threshold at 0.3 c/d (Fig. 3A). CLN6*^nclf^* mice also showed normal OKT thresholds up to four months of age. However, starting at five months of age, CLN6*^nclf^* mice appeared to have a significant and rapid decline in OKT thresholds with 0.05 c/d by eight months of age (Fig. 3A). The larger variability of OKT thresholds in older animals most likely reflects variable disease progression in different CLN6*^nclf^* mice.

To verify that the progressive decline in OKT readings and visual acuity was indeed due to vision loss and not motor-neuron deficits or cognitive difficulties, we performed rotarod experiments as three repeated trials per day with each mouse for two consecutive days (Fig. 3B). Four and six month old wild-type as well as CLN6*^nclf^* mice showed approximately equal rates of improvement in latency times, indicating that they learned equally well to stay longer on the rotarod on the second day of analysis. At eight months of age, wild-type animals also showed improved latency times whereas no enhancement was observed with eight month old CLN6*^nclf^* mice. This indicates a reduction in cognitive function and/or motor impairment in CLN6*^nclf^* mice later than 8 months of age. Together these results support the hypothesis that the decrease in OKT measurements from four to six months seen in CLN6*^nclf^* mice results from a loss of visual acuity and is not severely affected by motor problems.

To complement the OKT experiments, we performed dark adapted (scotopic) ERG measurements as an independent measure of retinal function. Rod photoreceptor function (a-wave) and inner retinal function (b-wave) were both determined for wild-type and CLN6*^nclf^* mice (Fig. 3 C–E). The a-wave amplitude significantly decreased in CLN6*^nclf^* mice starting at one month of age followed by the b-wave at three months of age (Fig. 3C). The amplitude loss of both components in CLN6*^nclf^* mice was even more pronounced at higher ages. As expected, the response amplitude of wild-type mice showed only a mild descent until the age of eight months.

### Early Stress Response and Inflammatory Marker Transcripts in Degenerating CLN6*^nclf^* Retinas

**Figure 4:**
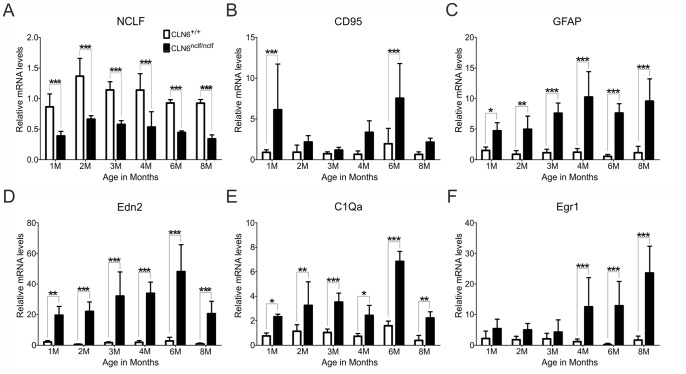
Early induction of stress response and glial marker transcripts in degenerating CLN6*^nclf^* retinas. A-F. Quantitative real-time RT-PCR expression analysis of CLN6*^nclf^* retinas compared to age-matched wild-type controls. Relative mRNA levels were analyzed for NCLF (A), CD95 (B), GFAP (C), EDN2 (D), C1Qa (E), and EGR1 (F). mRNA expression was normalized to the reference gene ATP5B and graphed relative to age-matched wild-type (± SD). *p<0.05; **p<0.01; ***p<0.001, n = 7–10 animals per age, two-way ANOVA followed by Bonferroni post test.

We next used quantitative real-time RT-PCR to address the question whether the progressive retinal degeneration in CLN6*^nclf^* mice can be associated with cell death, stress response and inflammation. First, NCLF mRNA levels were determined to study a potential nonsense-mediated decay of mutant mRNA. NCLF transcript levels were significantly reduced in CLN6*^nclf^* retinas (Fig. 4A) starting as early as one month of age indicating active ER-stress pathways in mutant cells. Also nonsense-mediated decay has been described in other NCL models [25] and the high expression of CD95 (alias Fas receptor) in our mouse model is indicative for constant apoptosis (Fig. 4B). The mRNA levels for GFAP in reactive Müller cells were increased five to ten fold in CLN6*^nclf^* mice at all ages (Fig. 4C) and correlates with our immunohistochemical analyses (above in Fig. 1B). Expression levels of the photoreceptor stress marker endothelin 2 (EDN2) and microglia markers complement C1q subunit a (C1qa) and early growth response 1 (EGR1) were also assessed. Both EDN2 and C1qa were strongly up-regulated in CLN6*^nclf^* retinas, indicating a prominent stress response at all ages (Fig. 4D–E). EGR1 transcript levels were also increased in CLN6*^nclf^* retinas but only as of four months of age. These experiments suggest that retinal degeneration in CLN6*^nclf^* retinas follows a temporally ordered sequence of very early cell stress and concomitant microglial response.

### Ramified Microglia and Improved Retinal Morphology in Curcumin and DHA-supplemented CLN6^nclf^ Mice

**Figure 5:**
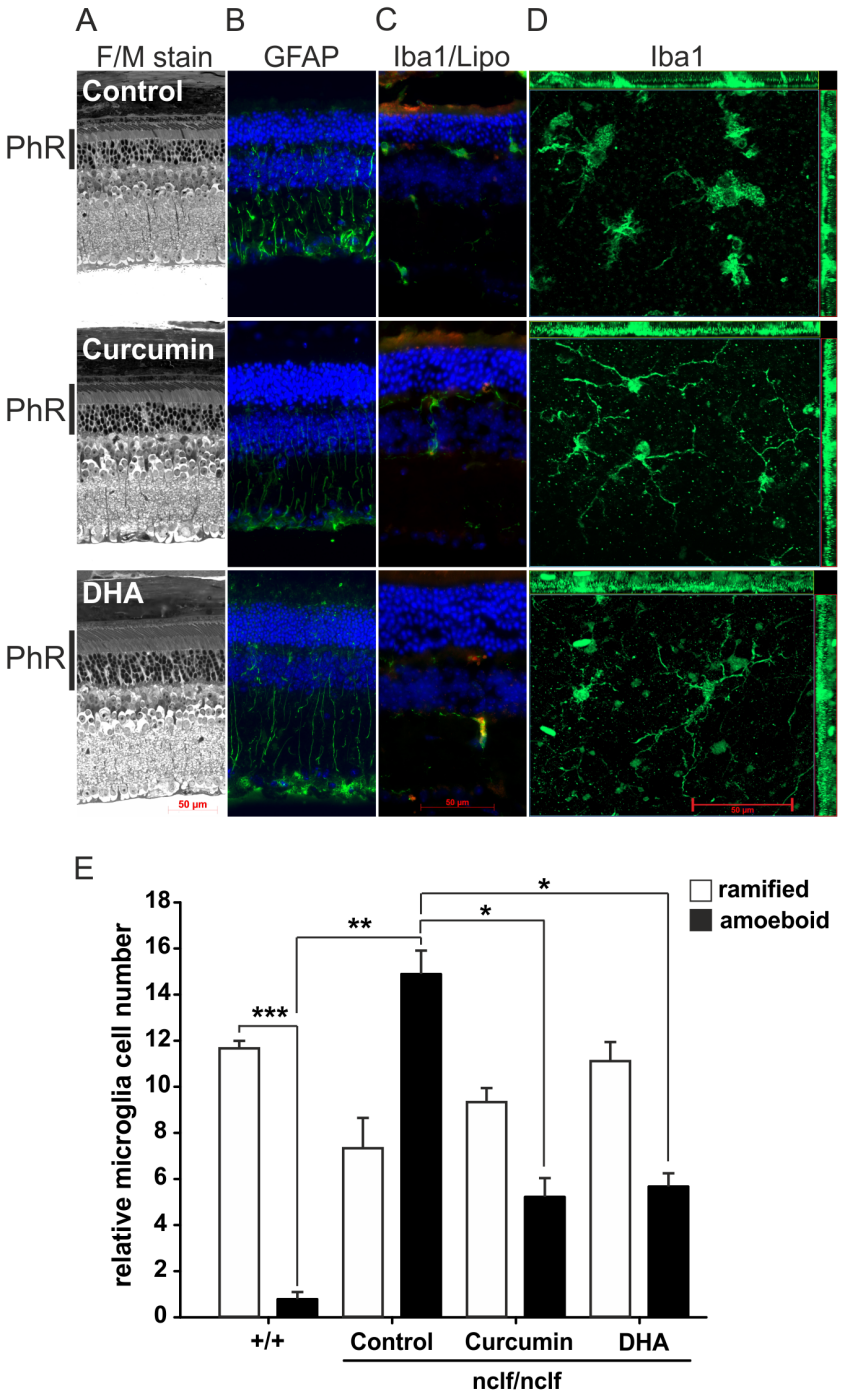
CLN6*^nclf^* mice supplemented with 0.6% curcumin or 5% DHA for 30 weeks after weaning display ramified retinal microglia. A. Histological comparison of control animals and food-supplemented retinas. B. Immunolabeling of Müller cells with anti-GFAP antibody C. Staining of microglial cells with anti-Iba1 antibody and detection of autofluorescent lipofuscin accumulation in CLN6*^nclf^* retinas. D. Anti-Iba1 labeled retinal flat mounts detect the morphology of microglia in control retinas and food-supplemented retinas. The thickness of the flat-mount is indicated on the sides of the image. Scale bar, 50 µm. E. Quantification of ramified and amoeboid microglial cells in nine independent image areas of three individual flat mounts (mean ± SEM). *p<0.05; **p<0.01; ***p<0.001, n = 3 animals per group, unpaired two-tailed *T*-test.

We next assessed whether a dietary supplementation of CLN6*^nclf^* mice with the immunomodulatory compounds curcumin and DHA could attenuate microglial reactivity and retinal degeneration. Three week old CLN6*^nclf^* mice received either control chow or chow supplemented with 0.6% curcumin or 5% DHA for 30 weeks starting directly after weaning. The histological comparison of cross sections revealed that DHA-supplemented retinas appeared to have a preserved structure of the photoreceptor layer (Fig. 5A, black bars), but the extent of Müller cell reactivity was not changed (Fig. 5B). The localization of microglia and lipofuscin deposits was also comparable in control-fed and supplemented mice (Fig. 5C). Exemplified flat mount analyses of microglial morphology then indicated that the amoeboid phenotype observed in control CLN6*^nclf^* mice was reduced in curcumin and DHA-treated animals compared to control-fed animals (Fig. 5D). We then performed a quantitative analysis of microglial cell numbers for the ramified and amoeboid phenotypes, respectively. We first noticed that the normal situation of mostly ramified cells in the retina of wild-type mice was completely reversed in control-treated CLN6*^nclf^* mice (Fig. 5E). The fraction of amoeboid cells was strongly increased in CLN6*^nclf^* mice and the presence of this cell population was significantly suppressed in curcumin and DHA-treated CLN6*^nclf^* retinas (Fig. 5E). Conversely, there was a tendency that the amount of ramified microglial cells was increased when CLN6*^nclf^* mice received supplementation with curcumin or DHA (Fig. 5E).

**Figure 6:**
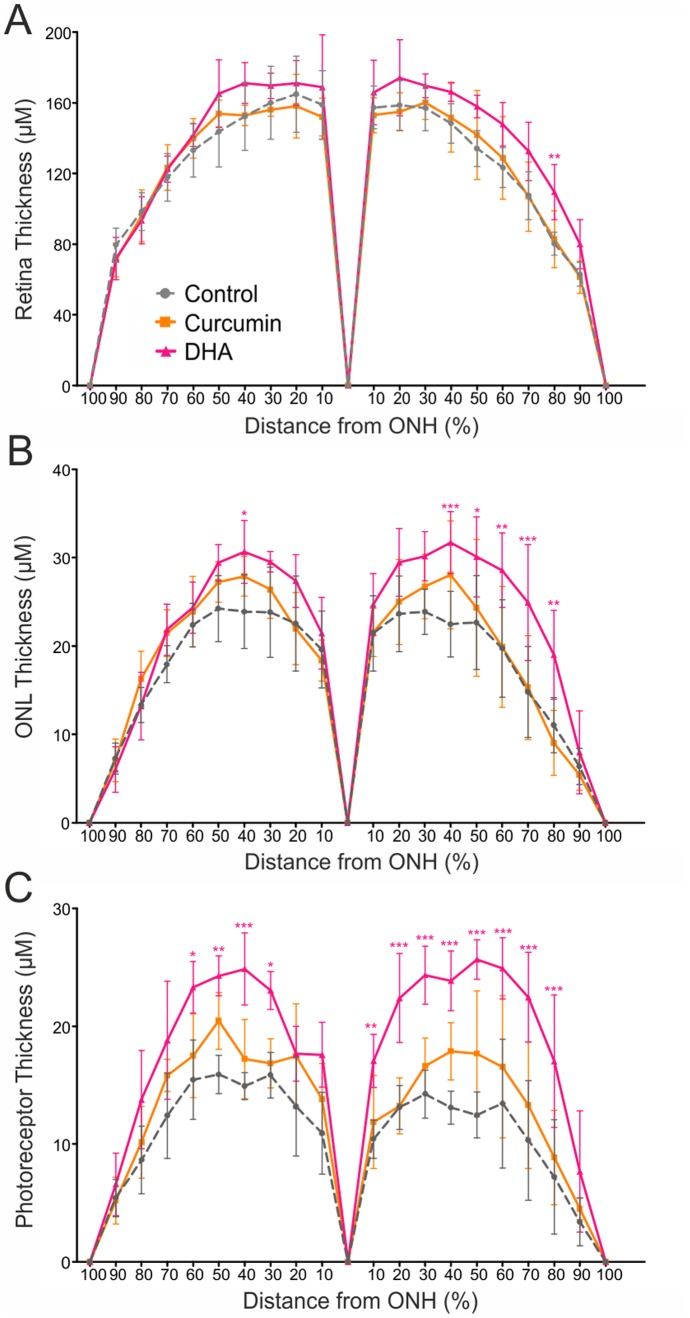
DHA-supplemented CLN6*^nclf^* retinas have a thicker ONL and photoreceptor layer. Anterior and posterior retinal areas were divided into ten sections with the optic nerve as reference. A. Quantification of whole retinal thickness of curcumin and DHA-supplemented versus control retinas (mean ± SD). B. Quantification of the ONL (mean ± SD). C. Quantification of the photoreceptor layer (mean ± SD) *p<0.05; **p<0.01; ***p<0.001 CLN6*^nclf^* vs. age-matched wild-type mice, n = 5 animals per age group, two-way ANOVA followed by Bonferroni post-test.

In our preliminary analysis we noted a potentially preserved retinal morphology in curcumin and DHA-supplemented CLN6*^nclf^* mice (Fig. 5A). To relate this observation with the applied treatment we performed a detailed quantitative analyses of the total retinal thickness, the outer nuclear layer thickness, and the photoreceptor layer thickness in all groups. Curcumin-supplemented retinas had approximately the same total thickness of the total retina, the ONL and the photoreceptor layer as control retinas (Fig. 6). In DHA-fed CLN6*^nclf^* mice, the total retinal thickness was not changed significantly (Fig. 6A), the ONL was significantly thicker in some regions of the retina (Fig. 6B) and the photoreceptor layer was thicker in most retinal regions analyzed (Fig. 6C). These data indicate that both curcumin and DHA affect the microglial network in CLN6*nclf* retinas but that only DHA leads to a significant preservation of the retinal structure.

### Improved Visual Function in Curcumin and DHA-supplemented CLN6^nclf^ Mice

**Figure 7:**
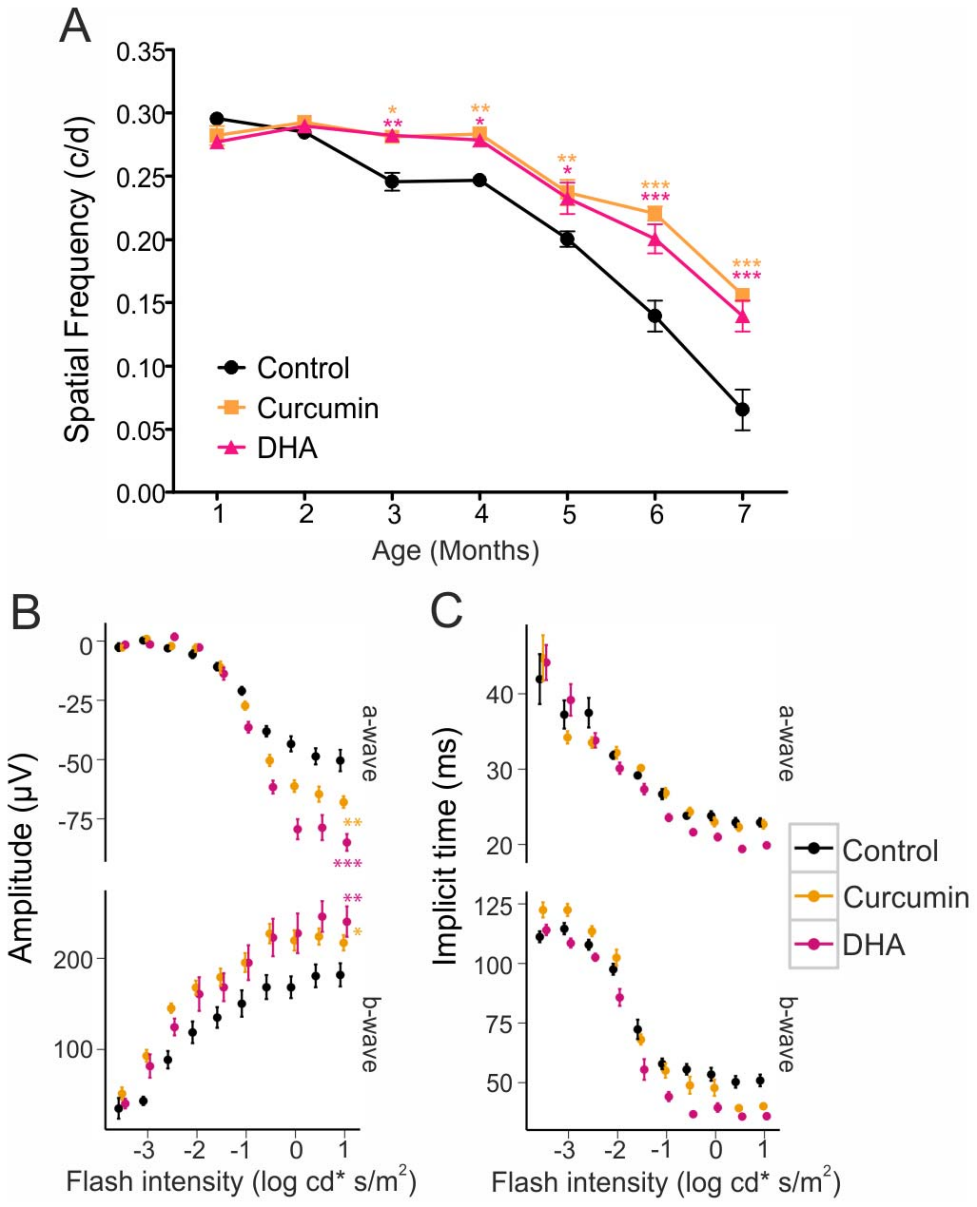
Curcumin and DHA-supplemented CLN6*^nclf^* mice have better visual acuity and ERG measurements compared to control CLN6*^nclf^* mice. A. Mice supplemented with curcumin and DHA had higher optokinetic tracking thresholds starting at 3 months of age (mean ± SD). *p<0.05; **p<0.01; ***p<0.001, n = 12 animals per age group, two-way ANOVA followed by Bonferroni post-test. B. Dark adapted ERG responses recorded at 7 months of age show higher a-wave amplitudes for higher flash intensities and higher b-wave amplitudes for almost all flash intensities for supplemented mice (two-way ANOVA for the amplitudes of the highest flash intensity: a-wave: *p* = 0.002 and *p*<0.0001, b-wave: *p* = 0.028 and *p* = 0.009 for curcumin and DHA, respectively). While a-wave implicit times did not differ for curcumin, the DHA implicit time was shorter for higher flash intensities. B-wave implicit times of responses to higher flash intensities were shorter for supplemented mice (a-wave: *p* = 0.77 and *p* = 0.0002, b-wave: *p* = 0.0004 and *p*<0.0001).

Dietary supplementation of CLN6*^nclf^* mice with curcumin and DHA resulted in significantly higher visual acuity compared to control CLN6*^nclf^* mice starting at three months of age (Fig. 7A). In addition, curcumin and DHA supplemented animals showed significantly higher amplitudes of ERG-responses recorded at seven months of age (Fig. 7B). This indicates a preservation of photoreceptor (a-wave) and inner retinal function (b-wave) (Fig. 7B). For DHA-treated animals, implicit times for a- and b-waves were shorter for higher flash intensities being consistent with less severe retinal degeneration (Fig. 7C).

## Discussion

Decline of visual perception is an early symptom in most forms of human NCL, indicating that the retina is highly vulnerable to NCL pathologies [6]. In this study, we carefully characterized the ocular phenotype in the well-established CLN6*^nclf^* mouse model of variant late infantile NCL. We found a progressive retinal degeneration along with gliosis and microglial reactivity which may contribute to disease progression. A supplementation study with curcumin and DHA changed the microglial phenotype to a less amoeboid and improved retinal function in CLN6*^nclf^* mice.

Initial studies done by Bronson et al. identified an accumulation of autofluorescent deposit and loss of cell layers in the ONL of the CLN6*^nclf^* [12]. Our morphometric histological data showed that the degeneration of the outer retina was preceding cell death in the inner retina, which implicates that photoreceptor cells were very early affected during disease progression. We also noticed early diminished ERG responses, which could reflect the retinal thinning and quantitative cell loss. However, whereas ERG responses were already impaired at one month of age, OKT threshold changes were only significant from five months of age onwards. The phenomenon of disconcordant electrophysiological and visual behavioral profiles has been recently described in mutant rhodopsin transgenic rats [26]. Our rotarod experiments showed that the locomotor function of CLN6*^nclf^* mice was relatively normal at four months of age but was significantly impaired at eight months of age. These data are in good agreement with a recent report that showed reduced motor function in CLN6*^nclf^* mice from 34 weeks of age onwards using rotarod and grip strength tests [27].

In contrast to the rapidly declining amplitudes of a- and the b-waves in CLN6*^nclf^* mice, CLN3*^Δex7–8^* animals, a model for juvenile NCL, have relatively normal scotopic ERGs until nine months of age, indicating a late onset retinal degeneration [28]. In the PPT1^−/−^ (CLN1) mouse model of infantile NCL, only moderate changes in ERG response were identified [29]. Therefore, we conclude that CLN6*^nclf^* mice show the most severe and progressive decline in visual function among the NCL mouse models tested so far.

Our analysis of Müller glia by GFAP staining and analysis of mRNA expression showed early activation already present at one month and progressively increasing with age. This is considerably earlier than GFAP staining of astrocytes in CLN6*^nclf^* cerebral cortices, which appeared between five to six months of age [12], [13]. Therefore, gliosis associated with neuronal degeneration in the CLN6*^nclf^* mouse seems to occur in the eye before the brain. Iba1 detection of microglial cells in retinal sections and flat-mounts demonstrated a mixed population of alerted microglia at one month of age which became a homogeneous group of amoeboid microglia by four months. Alerted microglial cells also contained autofluorescent granules at all ages examined, which could reflect phagocytic processes of dying neurons affected by lysosomal storage of ceroid lipofuscin. Studies in CLN6*^nclf^* and PPT1^−/−^ mutant mouse brain show that reactive microglia and astrocytes may induce localized damage in the brain [30]. Since lipofuscin deposits are equally distributed in the retina, these results suggest that lipofuscin is not the trigger for microglial cells. The trigger may rather be stress signals sent from neurons to the glial cells. Induction of the microglial markers C1Qa at all ages or EGR1 starting at four months were also accompanied by strong up-regulation of EDN2, a secreted factor of stressed photoreceptors [31]. We also identified increased expression of the apoptosis transcript marker CD95. However, tunnel stains of CLN6*^nclf^* retinas revealed one or two cells undergoing apoptosis at any given time further confirming the presence of low-grade progressive degeneration (data not shown).

Another interesting aspect of the pathology is the significantly reduced mRNA expression of CLN6 in the CLN6*^nclf^* retina. A 50% or more reduction of retinal CLN6 expression was noticed in our study at all time points analyzed and we hypothesized that this phenomenon reflects nonsense-mediated decay. Consistent with our data, Kanninen et al. recently identified a reduced but not absent expression of CLN6 in the CLN6*^nclf^* eye at 12 and 24 weeks of age, which was linked to an accumulation of biometals in the CNS [27]. 30–40% reduced CLN6 mRNA levels were also identified in the developing and adult brain of CLN6*^nclf^* mice [32] as well as in immortalized brain cells from young CLN6*^nclf^* animals [33]. A related observation of reduced CLN6 mRNA was made in the South Hampshire sheep model of CLN6 disease [34]. Of note, the same frame-shift mutation (c.307insC) is found in CLN6*^nclf^* mice and human CLN6 patients. Cell culture expression studies with mutant CLN6 revealed that the decrease in CLN6 transcripts caused a corresponding decrease in protein levels [35]. Nonsense-mediated decay has also been reported for CLN1, CLN2, and CLN3 and a potential therapeutic option could be treatment with read-through drugs that enhance protein function.

Studies done by Groh et al, in which lymphocytes were inactivated in PPT1^−/−^ mice, showed a substantial disease attenuation, unequivocally defining immune cells as pathogenic mediators in infantile NCL [15]. Moreover, pharmacological and genetic suppression of the immune system in the CLN3^−/−^ mouse model of juvenile NCL resulted in improved motor performance [36]. Since reactive microglia have also been previously identified in CLN6*^nclf^* sheep brain [14] as well as in human cortical biopsies from CLN3 patients [1], targeting the immune system and by extension, inflammation, could be one option for therapeutic intervention.

Several natural compounds exist which can target microglial pathways whilst supporting neuronal survival. Curcumin is a herbal medicine which has been used for centuries in India and China [37]. Curcumin has been shown to block the production of nitric oxide [17], to reduce the secretion of proinflammatory cytokines [18], and to protect dopaminergic neurons against microglia-mediated neurotoxicity [38]. Curcumin supplementation also showed functional and structural protection of photoreceptors against acute light damage in rats along with decreased inflammatory gene expression [39].

DHA is highly enriched in the retina and is a precursor for neuroprotectin D1, promoting the survival of photoreceptors and RPE cells [40]. Moreover, it has recently been shown that DHA can inhibit the synthesis of inflammatory products in microglia allowing better survival of neural progenitor cells [19]. Furthermore, it has been previously reported that patients with juvenile NCL have reduced DHA levels in the plasma and cerebral cortex, which may contribute to retinal and brain degeneration [41].

Based on these studies, CLN6*^nclf^* mice were supplemented with curcumin or DHA for 30 weeks immediately after weaning in order to reduce glial reactivity and promote neuronal survival. With both dietary regimens, OKT measurements were significantly higher compared to non-supplemented control mice starting at three months. ERG analysis also showed improvements in b-wave signals for both compounds with DHA having greater preservation of the a-wave. The preservation of the photoreceptor layer and particularly the outer segments in DHA-treated mice was also highlighted in morphometric and histological analyses. A beneficial effect on photoreceptor outer segments has also been seen in DHA supplementation of rhodopsin mutant rats, although no alteration in the rate of retinal degeneration was detected [42]. Iba1 staining of microglia showed significantly less amoeboid and alerted cells in both supplemented CLN6*^nclf^* retinas compared to control-fed CLN6*^nclf^* animals. It is important to note that microglia morphology is not always equivalent to the inflammation state [43]. However, the microglial phenotype identified in the supplemented CLN6*^nclf^* retinas looked similar to those found in DHA-supplemented retinoschisin-deficient mice [20]. In this retinal degeneration model, the microglial population produced less pro-inflammatory cytokines and the retinal morphology was improved upon DHA-treatment [20]. Thus, we speculate that DHA- and also curcumin-supplemented CLN6*^nclf^* retinas display less microglial reactivity.

Microglial involvement in neurodegenerative diseases such as Alzheimeŕs disease, Multiple Sclerosis and now NCL is becoming better understood. We conclude from our studies that targeting reactive microglia whilst supporting neuronal survival with derivatives of natural compounds or pharmaceuticals could have therapeutic potential. Since the retina is often affected earlier than the brain, analyses of the ocular phenotype in NCL is helpful to understand molecular mechanisms and could also be useful to develop diagnostic tools for experimental therapies.
