# Correction: Progressive Retinal Degeneration and Glial Activation in the CLN6*^nclf^* Mouse Model of Neuronal Ceroid Lipofuscinosis: A Beneficial Effect of DHA and Curcumin Supplementation

**DOI:** 10.1371/annotation/ae907882-62e0-4803-8c00-35b30a649fe9

**Published:** 2013-10-09

**Authors:** Myriam Mirza, Cornelia Volz, Marcus Karlstetter, Monica Langiu, Aleksandra Somogyi, Mika O. Ruonala, Ernst R. Tamm, Herbert Jägle, Thomas Langmann

There were errors in the author affiliations of Herbert Jägle and Thomas Langmann. The correct affiliations for these authors are:

Herbert Jägle: Department of Ophthalmology, University of Regensburg, Regensburg, Germany

Thomas Langmann: Department of Ophthalmology, University of Cologne, Cologne, Germany 

